# Programmable optical filters based on feed-forward photonic meshes

**DOI:** 10.1038/s41377-026-02461-9

**Published:** 2026-08-19

**Authors:** Carson G. Valdez, Anne R. Kroo, Marek Vlk, Charles Roques-Carmes, Shanhui Fan, David A. B. Miller, Olav Solgaard

**Affiliations:** 1 Stanford University, Ginzton Laboratory, 348 Via Pueblo Mall, Stanford, CA USA; 2 Department of Physics and Technology, UiT The Arctic University of Norway, Tromsø, Norway

**Keywords:** Integrated optics, Silicon photonics, Nanophotonics and plasmonics, Near-infrared spectroscopy, Photonic devices

## Abstract

We demonstrate an integrated photonic circuit based on feed-forward photonic meshes that may be reconfigured to perform a wide range of spectral filter functions in a programmable way. We investigate a subset of the available filter functions, demonstrating that a ${\rm{N}}=4$ input triangular mesh with ${\rm{M}}=3$ layers may be operated via self-configuration algorithms to filter M arbitrary wavelengths from a given input spectrum. The tunable nature of the architecture enables preconfigured filter functions to be swept in the spectral domain continuously over the free spectral range of the device. This removes any strict requirements between the design parameters of the architecture and the center wavelength of a desired filter function. With this architecture, we experimentally demonstrate arbitrary wavelength rejection filters with contrasts as deep as 40 dB. Further, by intentionally selecting the center wavelengths of each filter function to lie along a wavelength grid defined by $\Delta {\rm{\lambda }}={{\rm{\lambda }}}_{{\rm{fsr}}}/{\rm{N}}$ we demonstrate deep wavelength division demultiplexing (DWDM) with inter-channel crosstalk between −25 dB and −40 dB. Unlike typical DWDM systems, in this architecture the center wavelength of each channel is not fixed at fabrication and instead may be programmatically defined. This device demonstrates advantages over typical methods for DWDM, Raman spectroscopy, and correlation spectroscopy as well as other applications.

## Abstract (web summary)

We demonstrate a photonic mesh architecture capable of implementing a wide variety of optical filter functions including bandpass filters, notch filters, and wavelength division demultiplexers, all with programmable center wavelengths.

## Graphical abstract

**Figure:**
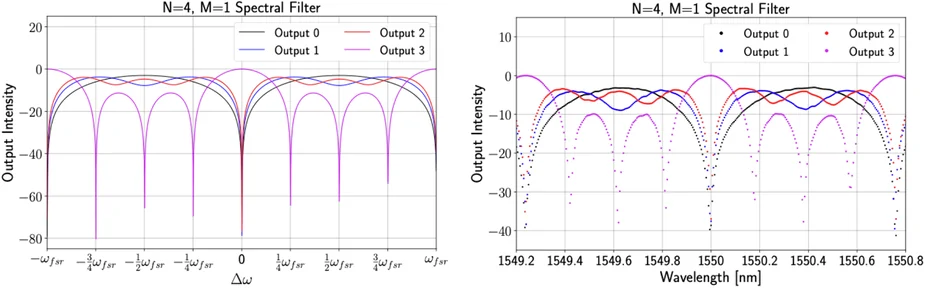

## Introduction

Spectral filters are ubiquitous components used in nearly every optical application spanning telecommunications^1,2^, environmental sensing^3,4^, astrophysics^5,6^, and biomedical sensing^7^. Integrated spectral filters such as arrayed waveguide gratings^8,9^ and distributed Bragg reflectors^10,11^ have long offered a path toward miniaturization. Employing additional local heating elements to shift the spectral band of these devices has provided a degree of tunability^12,13^; however, this method cannot change the spectral profile of the device.

Traditional fixed-function photonic filters offer high performance but lack flexibility, requiring separate device designs and fabrication runs for each target function. Programmable filters enable a single hardware platform to implement a wide range of spectral responses, enabling further applications including all-optical signal processing^14^, correlation spectroscopy^15^, and wavelength tunability for external cavity lasers^16^. Programmable spectral filters have been realized utilizing a number of recirculating mesh architectures^14,17–19^. In these systems, light is routed through closed-loop paths that provide spectral selectivity via interference and resonance effects.

Feed-forward-based photonic meshes may be operated using progressive optimization algorithms that enable distinct functionalities that have been widely demonstrated for spatial mode processing^20–24^. However, these architectures are typically designed for broadband operation, lacking the strong wavelength dependence necessary to generate spectral filters.

Recently, we have laid the analytical groundwork for a photonic integrated circuit (PIC) architecture based on feed-forward photonic meshes capable of performing arbitrary filter functions across an available design space^25^. Here we experimentally demonstrate that such a device is capable of filtering multiple arbitrary wavelengths while being programmed via a self-configuration algorithm. Such an algorithm automatically adapts a layer of the mesh to reject an arbitrarily given wavelength. Each layer of the photonic mesh is capable of filtering an additional independent wavelength from its respective outputs. This programmable nature of the photonic mesh enables filter functions to be dynamically adjusted post-fabrication.

We demonstrate a subset of the available filter functions which may be implemented by this architecture. From among the possible filter functions, we demonstrate a bandpass filter whose center wavelength may be continuously tuned across a free spectral range. We implement a series of notch filters, demonstrating that multiple arbitrary wavelengths may be rejected from the outputs of the mesh with a contrast as deep as 40 dB. By choosing the filtered wavelengths to be in close proximity to each other, we are able to form a wideband rejection filter spanning a large portion of the free spectral range. Further, by choosing the filtered wavelengths to correspond to a designed wavelength grid, we perform wavelength division demultiplexing. Unlike typical DWDM systems whose wavelength grid is determined at fabrication, the wavelength grid of this architecture may be determined by self-configuration algorithms and adjusted dynamically.

## Results

### Theory and modeling

**Fig. 1:**
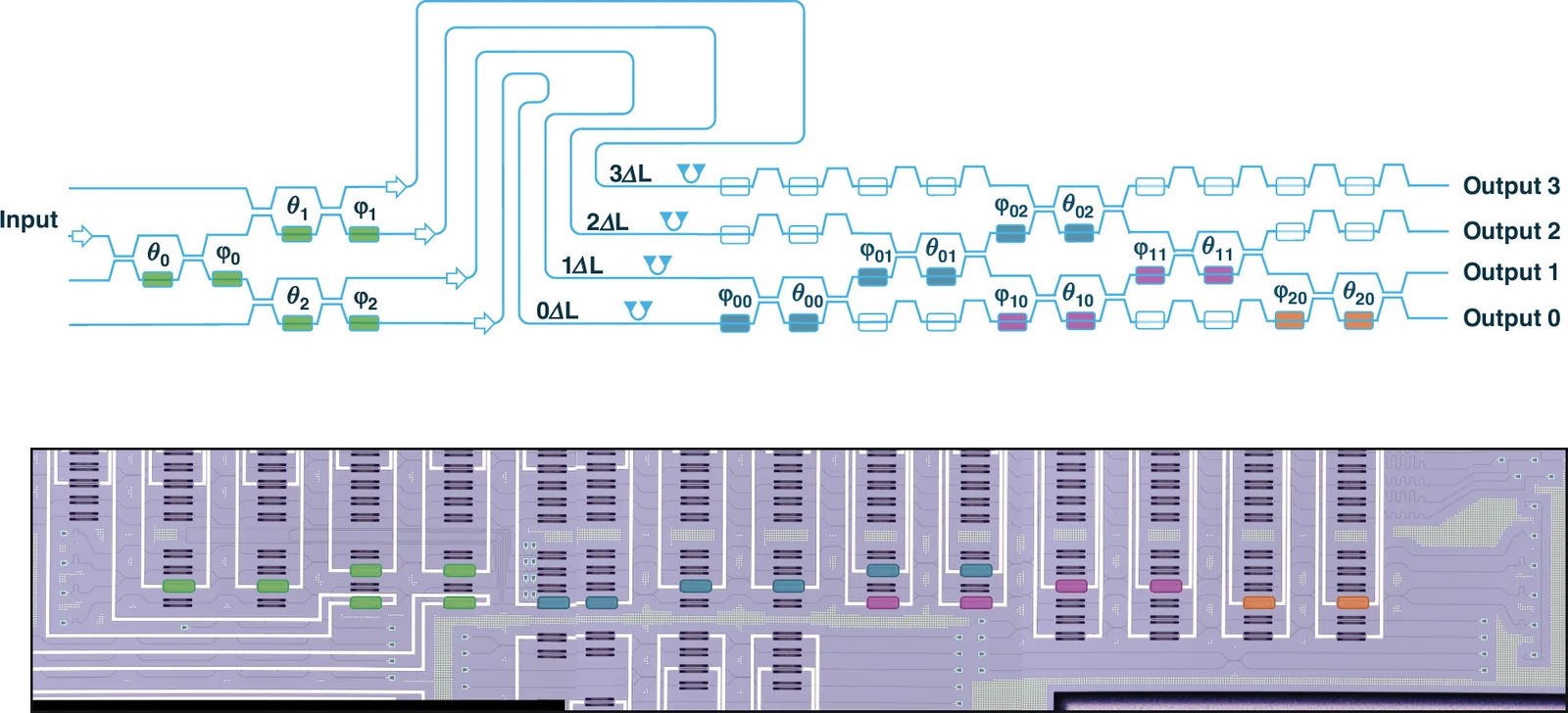
(Top) Schematic diagram of a four-channel programmable spectral filter. (Bottom) Microscope image of the fabricated photonic integrated circuit. The photonic circuit is comprised of three subcircuits: a power splitter, an array of waveguide delay lines, and a photonic mesh. Here, the power splitting subcircuit has been implemented via a two-stage binary tree of balanced MZIs (green). The array of *N* waveguide delay lines introduces the phase shifts ${{\rm{\phi }}}_{p}$ to each respective waveguide. The array of waveguide delay lines has been designed with a uniform increment $\Delta {\rm{L}}=740{\rm{\mu }}{\rm{m}}$ to introduce a linear phase tilt. The photonic mesh applies a programmable matrix transformation *O*, described by matrix elements ${{\rm{O}}}_{{pq}}$. We implement this subcircuit as an N=4, M=3 triangular mesh capable of implementing any linear unitary 4×4 matrix transformation, where N represents the number of input channels, and M represents the independent diagonal self-configuring line layers of the mesh (blue, purple, orange)

The architecture of this programmable filter consists of three subcircuits as displayed in Fig. 1: a $1\times {\rm{N}}$ arbitrary power splitter, an array of N waveguide delay lines, and an ${\rm{M}}\times {\rm{N}}$ programmable photonic mesh. The power splitting subcircuit is responsible for distributing the input optical field into a set of parallel channels. This operation creates multiple coherent replicas of the input signal, each of which will subsequently experience a distinct temporal delay before being recombined and processed by the photonic mesh. In this sense, the power splitting network establishes the dimensionality of the system by defining the number of available delay taps. The array of waveguide delay lines forms the core physical mechanism by which spectral information is mapped into a vector space suitable for processing by the photonic mesh. Following the programmable power splitting stage, each branch of the input field propagates through a waveguide of distinct optical path length, thereby accumulating a unique frequency-dependent phase shift. This operation converts variations in optical frequency into structured phase differences across the set of channels. The programmable photonic mesh constitutes the final and most powerful stage of the spectral processing architecture. Having mapped the continuous optical spectrum onto a discrete vector of complex amplitudes via the power splitting subcircuit and the array of waveguide delay lines, the role of the photonic mesh is to implement arbitrary linear, unitary transformations on this vector through coherent interference.

A key requirement of the programmable photonic mesh is that the chosen topology supports self-configuration algorithms. From among the variety of established photonic mesh topologies, the binary tree, diagonal line, and triangular topologies are well suited for this reason. In this work, we have chosen to use an N-element triangular mesh, which itself can be treated as M = N-1 independent diagonal line layers. This partitioning of the triangular mesh into independent functional layers is highlighted in Fig. 1, where the phase shifters associated with independent layers have been color-coded as blue, purple, and orange. Each diagonal line layer may be independently operated as a self-aligning beam coupler, interferometrically combining a coherent beam represented by a vector of complex amplitudes at the layer-associated inputs to a single output.

In this section, we derive the transfer function of such an architecture, similar to ref. ^25^, albeit with a different choice of reference waveguide and indexing system to closely match the physical implementation of the architecture developed in this work. For the analysis of this system, we assert the simplifying assumption that over a reasonable bandwidth, the operations of both the power splitting and photonic mesh subcircuits are wavelength independent. This means that any wavelength dependence of the system results entirely from path length differences introduced in the array of waveguide delay lines. In a practical implementation, this condition is satisfied to first order by ensuring that all possible paths through these subcircuits are of equal length, as shown in Fig. 1.

The power splitting subcircuit divides a single input between ${\rm{N}}=4$ output waveguides with programmable splitting ratios such that the output complex fields at each waveguide may be adjusted dynamically. The complex field at each output of the power splitter is given by ${{\rm{a}}}_{p}$. We assume that the device is lossless such that the input power is conserved as the sum of all output powers. However, a constant overall loss in the circuit may be treated as a pre-factor without affecting the following spectral analysis. This is expressed through Eq. 1 where the variable *p* serves as an index of the ${\rm{N}}=4$ output waveguides of the power splitting subcircuit.

1 $$\mathop{\sum }\limits_{p=0}^{N-1}{\left|{a}_{p}\right|}^{2}=1$$

The 1×4 power-splitting subcircuit has been implemented via a two-stage binary tree of balanced Mach-Zehnder Interferometers, which have internal paths with equal length^21^. Each MZI is composed of a directional coupler acting as a nominal 50:50 beam splitter, a thermo-optic phase shifter ${{\rm{\theta }}}_{i}$ that controls the splitting ratio of each MZI, a second directional coupler acting as a nominal 50:50 beam combiner, and another thermo-optic phase shifter ${{\rm{\phi }}}_{i}$ that controls the relative phase between the two outputs of the MZI. Adjusting power to each of the 6 thermo-optic phase shifters in the binary tree enables arbitrary control of the complex splitting ratios between the ${\rm{N}}=4$ outputs of the power splitting subcircuit^23^.

The ${\rm{N}}=4$ outputs of the power splitting subcircuit serve as inputs to the subsequent array of waveguide delay lines. Each of the delay lines is a single-mode waveguide with an identical cross-section ($500\,{\rm{nm}}\times 220\,{\rm{nm}}$) and distinct lengths ${{\rm{l}}}_{p}$, given by Eq. 2.

2 $${l}_{p}={l}_{0}+\Delta {l}_{p}={l}_{0}+{k}_{p}\Delta l$$

Here ${{\rm{l}}}_{0}$ is the common waveguide length between all delay lines in the array, and ${{\rm{k}}}_{p}$ is a set of N integers that multiply a unit length $\Delta {\rm{l}}$ to determine the length increment between waveguide delays. The value $\Delta {{\rm{l}}}_{p}$ then represents the path length difference between the ${{\rm{p}}}^{{th}}$ and initial $({\rm{p}}=0)$ waveguide delays.

For a given waveguide geometry and material system, the effective refractive index ${{\rm{n}}}_{r}=\frac{{\rm{c}}{\rm{\beta }}({\rm{\omega }})}{\omega }$ of the propagating mode will have an explicit wavelength dependence, influenced by both material dispersion and waveguide dispersion. We model both components of the dispersion by a single term $\frac{{\rm{\delta }}{{\rm{n}}}_{r}}{\delta \omega }$. For an angular frequency of interest ${\rm{\omega }}={{\rm{\omega }}}_{0}+\Delta {\rm{\omega }}$ the effective refractive index is given by the linear approximation Eq. 3 where ${{\rm{\omega }}}_{0}$ is the center frequency and $\Delta {\rm{\omega }}$ is a frequency offset term.

3 $${n}_{r}\left(\omega \right)={n}_{r}\left({\omega }_{0}\right)+\Delta \omega \frac{\delta {n}_{r}}{\delta \omega }$$

The phase delay of the ${{\rm{p}}}^{{th}}$ waveguide relative to the initial waveguide $({\rm{p}}=0)$ may be expressed in terms of the propagation constant and relative delay length of each waveguide:

4 $$\Delta {\phi }_{p}\left(\Delta \omega \right)=\beta \left(\Delta \omega \right)\Delta {l}_{p}=\left[{n}_{r}\left({\omega }_{0}\right){\omega }_{0}+{n}_{g}\Delta \omega \right]\frac{\Delta {l}_{p}}{c}$$

where ${{\rm{n}}}_{g}$ denotes the group index of the waveguide delay lines. Expression 4 may be simplified further under the condition that, at a central angular frequency ${{\rm{\omega }}}_{0}$, the relative phase between each output of the arrayed waveguides is an integer multiple of $2{\rm{\pi }}$. This condition constitutes a flat wavefront at the output of the array of waveguide delay lines and may be achieved by designing the unit length $\Delta {\rm{l}}$ to satisfy Eq. 5 for a particular central frequency, where ${{\rm{z}}}_{p}$ is an integer multiple of 2π.

5 $$\frac{{n}_{r}\left({\omega }_{0}\right){\omega }_{0}\Delta {l}_{p}}{c}=2\pi {z}_{p}$$

Under these circumstances, the relative phase between delay lines as a function of the wavelength detuning is given by Eq. 6:

6 $$\Delta {\phi }_{p}\left(\Delta \omega \right)=\frac{{n}_{g}\Delta \omega \Delta {l}_{p}}{c}$$

Similarly to the unit length, we can define a unit time delay for the system based on the group index of the guided mode.

7 $$\Delta t=\frac{{n}_{g}\Delta l}{c}$$

By substituting Eqs. 2 and 7 into Eq. 6 we can further simplify the relative phase delay between waveguides such that it is a simple function of the unit time delay and angular frequency detuning relative to the nominal center frequency of the design. Note that the common ${{\rm{l}}}_{0}$ term has been dropped from Eq. 2 as the ${\rm{p}}=0$ waveguide delay line serves as a reference for all values $\Delta {{\rm{\phi }}}_{{\rm{p}}}$.

8 $$\Delta {\phi }_{p}\left(\Delta \omega \right)={k}_{p}\Delta \omega \Delta t$$

As shown in Fig. 1, the outputs of the waveguide delay lines serve as the direct inputs to the ${\rm{M}}\times {\rm{N}}$ photonic mesh of interconnected Mach-Zehnder Interferometers. In this work, we employ a ${\rm{N}}=4$ input triangular mesh. The four input triangular photonic mesh is implemented via ${\rm{M}}=3$ diagonal lines of balanced MZIs, each diagonal line with one fewer MZI than the previous. It has been well established^20,26,27^ that by tuning each of the ${\theta }_{{mp}}$ and ${{\rm{\phi }}}_{{mp}}$ phase shifters within the mesh, it may be configured to implement any linear, unitary 4×4 matrix operation represented by the matrix operator O, with elements ${{\rm{O}}}_{{qp}}$.

The MZIs used in the photonic mesh are nominally identical to those used in the power-splitting subcircuit. We have chosen to use balanced MZIs in both subcircuits as the nominally equal internal path lengths ensure, to first order, that their operations are wavelength independent. To ensure an identical propagation length through every potential path through the mesh, we have implemented dummy MZI bends as well as dummy phase-shifting elements which serve no function other than path-length and propagation loss matching. These additional elements help to ensure that any significant wavelength dependence of the PIC can be attributed to the arrayed waveguide delay lines.

For a photonic mesh represented by the matrix elements ${{\rm{O}}}_{{qp}}$, the transfer function to each output of the programmable spectral filter is given by Eq. 9 where ${\rm{x}}(\Delta {\rm{\omega }})$ is the input spectrum to the PIC and ${{\rm{y}}}_{q}(\Delta {\rm{\omega }})$ is the spectrum at the ${{\rm{q}}}^{{th}}$ output.

9 $${y}_{q}\left(\Delta \omega \right)=\mathop{\sum }\limits_{p=0}^{N-1}{a}_{p}{O}_{{qp}}{e}^{-j{k}_{p}\Delta \omega \Delta t}x\left(\Delta \omega \right)$$

This expression shows that the phase term will oscillate periodically as a function of the frequency detuning. From this periodicity, we can define the free spectral range (FSR) of the transfer function, which will be solely dependent on the group index and unit length difference of the waveguide delay lines.

10 $${\omega }_{{fsr}}=\frac{2\pi }{\Delta t}\to {f}_{{fsr}}=\frac{c}{{n}_{g}\Delta {l}_{0}}$$

Within a single FSR, the transfer function is determined entirely by the reconfigurable power splitting ratios ${{\rm{a}}}_{{\rm{p}}}$, the fixed relative delay lengths between the inputs of the photonic mesh ${{\rm{k}}}_{{\rm{p}}}$, and the reconfigurable matrix representation of the mesh itself ${{\rm{O}}}_{{qp}}$. Meanwhile, the minimum bandwidth of the generated transfer function is determined by the highest-order Fourier component used to synthesize the transfer function, given by ${c}_{0}$/$({n}_{g}\Delta {l}_{0}$N). This demonstrates that the sharpness of the available filter functions is inversely proportional to the longest pathlength difference of the system. In the limit of a large N system, this architecture can generate arbitrary filter functions within a single FSR.

As can be seen in Fig. 1, each diagonal line layer of the photonic mesh has a single output, which is directly routed to the output of the PIC. The remaining outputs from each layer form the inputs to the subsequent diagonal line of the mesh. Each layer is capable of performing an independent filter function on the inputs of that layer. This is such that an ${\rm{N}}=4$ spectral filter may perform as many as ${\rm{N}}-1=3$ independent filter functions.

For an arbitrarily configured transfer function or set of transfer functions, the center frequency of the programmable filter may be dynamically tuned by introducing an additional, wavelength-independent phase profile to the inputs of the photonic mesh. This additional phase front does not rely on the introduction of any new components to the generalized architecture. Instead, the existing phase profile may be augmented using programmable phase-shifting elements either in the first layer of the mesh or in the power splitting subcircuit. By introducing an additional phase profile ${{\rm{e}}}^{-j2\pi \gamma p}$, a preconfigured transfer function may be shifted in the frequency domain by ${\rm{\gamma }}{{\rm{\omega }}}_{{fsr}}$ where ${\rm{\gamma }}$ is a fraction of the FSR to be shifted. This functionality removes strict requirements on the design of the arrayed waveguide delay lines with respect to a desired center frequency.

11 $${y}_{q}\left(\Delta \omega \right)=\mathop{\sum }\limits_{p=0}^{N-1}{a}_{p}{O}_{{qp}}{e}^{-j{(k}_{p}\Delta \omega \Delta t-2\pi \gamma )}x\left(\Delta \omega \right)$$

### Design and Implementation

In this section, we further investigate a subset of the general architecture described above by enforcing three conditions. First, we require that the power splitter introduces a uniform power distribution between the N waveguide delay lines. Second, the waveguide delay lines are designed to introduce a linear phase tilt across the output waveguides. Third, the matrix elements of the photonic mesh are determined via a self-configuration algorithm^21–23^.

Given that we have assumed lossless components, the field strengths output from the power splitting subcircuit are given by:

12 $$\left|{a}_{p}\right|=\frac{1}{\surd N}$$

This condition may be satisfied through a calibration procedure for the MZI elements of the power-splitting subcircuit. This calibration procedure relies upon waveguide monitor taps which have been introduced following the array of waveguide delay lines. Each monitor employs a directional coupler designed to tap a small portion (3%) of the power from a bus waveguide. These directional couplers feed directly into a grating coupler designed to emit the tapped power at an angle near perpendicular to the surface of the PIC. The emitted power from each waveguide tap may be monitored while the parameters of the power-splitting circuit are swept, enabling calibration of the two-stage binary tree.

To begin the calibration procedure, a pilot source is launched into a root waveguide of the binary tree at the center wavelength of interest. With a constant input optical power, the following procedure is used to generate a mapping between input heater power and power splitting ratios:

1. Heater power to the ${{\rm{\theta }}}_{1}$ phase shifter is swept while monitoring the power transmitted to the ${\rm{p}}=2$ and ${\rm{p}}=3$ waveguide taps.
2. Heater power to the ${{\rm{\theta }}}_{2}$ phase shifter is swept while monitoring the power transmitted to the ${\rm{p}}=0$ and ${\rm{p}}=1$ waveguide taps.
3. Heater power to the ${{\rm{\theta }}}_{0}$ phase shifter is swept while monitoring the sum of powers transmitted to the ${\rm{p}}=0$ and ${\rm{p}}=1$ waveguide taps and the sum of powers transmitted to the ${\rm{p}}=2$ and ${\rm{p}}=3$ waveguide taps.

**Fig. 2:**
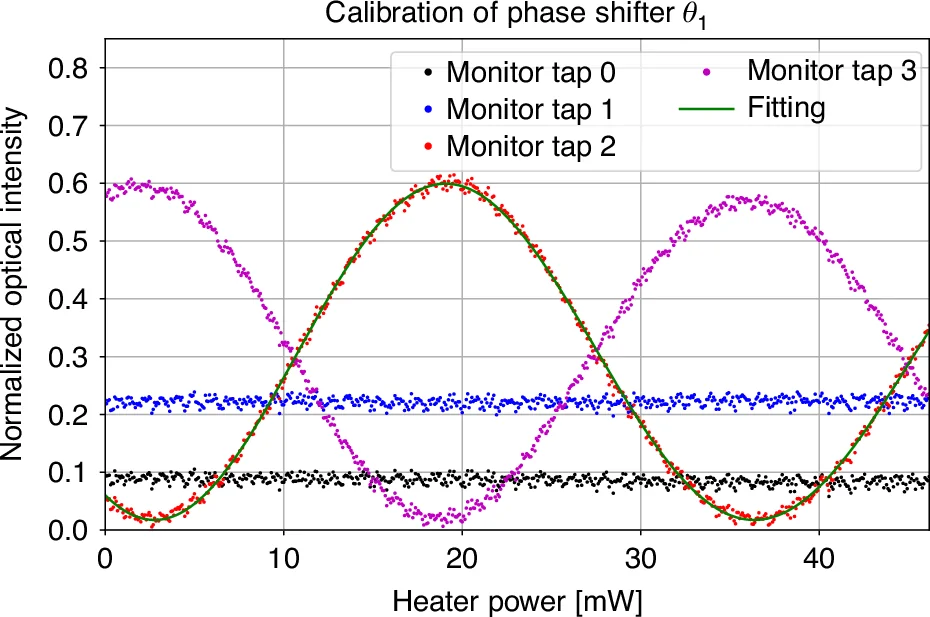
Calibration data of the ${{\rm{\theta }}}_{1}$ phase shifter at a central wavelength ${{\rm{\lambda }}}_{0}=1550\,{\rm{nm}}$. The ${{\rm{\theta }}}_{1}$ phase shifter controls the split ratio to the ${\rm{p}}=2$ and ${\rm{p}}=3$ input waveguides of the photonic mesh and has no impact on the ${\rm{p}}=1$ and ${\rm{p}}=0$ input waveguides. We observe the characteristic sinusoidal transmission of a balanced MZI and measure a ${{\rm{P}}}_{\pi }$ of 18 mW

An example of the monitored power through the waveguide taps is given in Fig. 2. By fitting the monitored power to the characteristic ${\sin }^{2}({\rm{\theta }})$ transmission function of an MZI, we are able to calibrate the heater power versus phase relationship of each thermo-optic phase shifter. Applying this mapping enables the subcircuit to arbitrarily and dynamically adjust splitting ratios. We record a ${{\rm{P}}}_{\pi }$, the heater power required to drive a ${\rm{\pi }}$ phase shift, of 18 mW which is in good agreement with similar thermo-optic phase shifters demonstrated on SOI platforms^28^.

To achieve a linear phase front at the output of the array of waveguide delay lines, each consecutive delay line is designed with an identical length increment.

13 $${k}_{p}=p$$

The slope of the phase front introduced by these delay lengths is determined by the frequency detuning parameter $\Delta {\rm{\omega }}$. At the designed center frequency, $\Delta {\rm{\omega }}=0$, the output of the waveguide delay lines will be a perfectly flat phase profile. For a negative frequency detuning, $\Delta {\rm{\omega }} < 0$, the delay lines will introduce a negative phase tilt. Conversely, for a positive frequency detuning, $\Delta {\rm\omega } > 0$, the delay lines will introduce a positive phase tilt.

The single-mode waveguide used throughout the architecture has a cross-section that is 220 nm tall by 500 nm wide. The fundamental quasi-TE mode has an effective and group index of ${{\rm{n}}}_{{\rm{r}}}=2.459$ and ${{\rm{n}}}_{{\rm{r}}}=2.459$, respectively, at a central wavelength of 1550 nm. These values have been simulated using the open-source Eigenmode and FDTD solver EMopt.

Based on the modeled group index and Eq. 10, a unit length ${\rm\Delta }{\rm{l}}=740\,{\rm\mu }{\rm{m}}$ has been chosen to achieve an FSR of 100 GHz. This enables an optimal four-channel frequency grid with a 25 GHz channel spacing, one of the common International Telecommunication Union (ITU) spacings for use in Dense Wavelength Division Multiplexing.

The final condition of this subset requires that the matrix elements of the photonic mesh are determined via a self-configuration algorithm^29^. To initialize this algorithm, each element of the mesh is first programmed to the transparent bar state, such that the outputs of the mesh are equal to the inputs. This condition is satisfied when each MZI element of the mesh has been configured to represent an identity matrix:

14 $${\rm{Transparent}}\,{\rm{Bar}}\,{\rm{State}}:\mathop{\to }\limits_{v}=\left[\begin{array}{cc}1 & 0\\ 0 & 1\end{array}\right]\mathop{\to }\limits_{u}$$

Initializing each MZI in the mesh to the transparent bar state is necessary for a photonic mesh that operates with only external detectors, as it allows for direct detection of the outputs for each MZI in the mesh. This is critical for the subsequent self-calibration procedure to treat each layer independently. Additional integrated photodetectors may be introduced at the outputs of each MZI to achieve the same effect, making the calibration procedure unnecessary, albeit at the expense of additional circuit complexity.

The transparent bar states may be achieved through the following progressive calibration algorithm for each of the ${{\rm{\theta }}}_{{mp}}$ phase shifters in the absence of internal photodetectors as follows^22,23^:

1. Using the calibrated power splitter, all input optical power is directed to the ${\rm{p}}=0$ input waveguide of the triangular photonic mesh.
2. Heater power to the ${{\rm{\theta }}}_{02}$ phase shifter is swept while monitoring the power transmitted to the ${\rm{q}}=3$ output waveguide.
3. The phase shifter ${{\rm{\theta }}}_{02}$ is set to the transparent cross state, which transmits light from the bottom input of the MZI to the top output:

15 $${\rm{Transparent}}\,{\rm{Cross}}\,{\rm{State}}:\mathop{\to }\limits_{v}=\left[\begin{array}{cc}0 & 1\\ 1 & 0\end{array}\right]\mathop{\to }\limits_{u}$$

1. Steps 1 through 3 are then repeated for phase shifters ${{\rm{\theta }}}_{01}$ and ${{\rm{\theta }}}_{00}$, progressively completing calibration of the first layer of the mesh.
2. Each element in the ${\rm{m}}=0$ diagonal line is set to the transparent bar state, effectively adapting the ${\rm{N}}=4$, ${\rm{M}}=3$ triangular mesh to a smaller ${\rm{N}}=3$, ${\rm{M}}=2$ triangular mesh.
3. Steps 1 through 4 are iterated for each progressively smaller triangular mesh until every element has been calibrated.

Following completion of the calibration stages, the power splitting subcircuit is set to project a uniform illumination across the N inputs of the photonic mesh. Further, each MZI element of the triangular mesh has been set to the transparent bar state as an initial condition of the self-configuration procedure. To program the filter characteristics, we use a self-configuration algorithm that requires a set of M narrow-linewidth pilot sources. Each of the M pilot sources is used to configure the filter function to the corresponding ${{\rm{m}}}^{{th}}$ layer of the mesh. Assuming that the first pilot source has an angular frequency ${{\rm{\omega }}}_{0}$, the input vector to the mesh, $\mathop{\to }\limits_{{u}_{p}}$, will have a flat phase profile and a uniform intensity distribution as given by expressions 5 and 12, respectively.

To configure the first layer of the mesh, each MZI is progressively optimized to maximize power out of the top-most waveguide of the associated layer^21^. This process automatically programs each element to collect all incoming light from the pilot source to the ${\rm{q}}={\rm{N}}-1-{\rm{m}}$ output waveguide. The output spectrum at the same waveguide may be calculated by applying the conditions of a uniform illumination, a linear phase tilt, and a matrix operator determined via self-configuration to the analytical solution of the general architecture, resulting in Eqs. 16 and 17 Here, the phase term ${\rm{\alpha }}$ represents the sum of the static and programmable phase terms: ${\rm{\alpha }}=\Delta {\rm{\omega }}\Delta {\rm{t}}+2{\rm{\pi }}{\rm{\gamma }}$.

16 $$y\left(\Delta \omega \right)=\frac{1}{N}[\mathop{\sum }\limits_{p=0}^{N-1}{e}^{-{jp}\alpha }x\left(\Delta \omega \right)$$

17 $$y\left(\Delta \omega \right)={e}^{-j\frac{\alpha }{2}\left(N-1\right)}\frac{{sinc}\left(N\frac{\alpha }{2}\right)}{{sinc}\left(\frac{\alpha }{2}\right)}x\left(\Delta \omega \right)$$

### Spectral filter functions

**Fig. 3:**
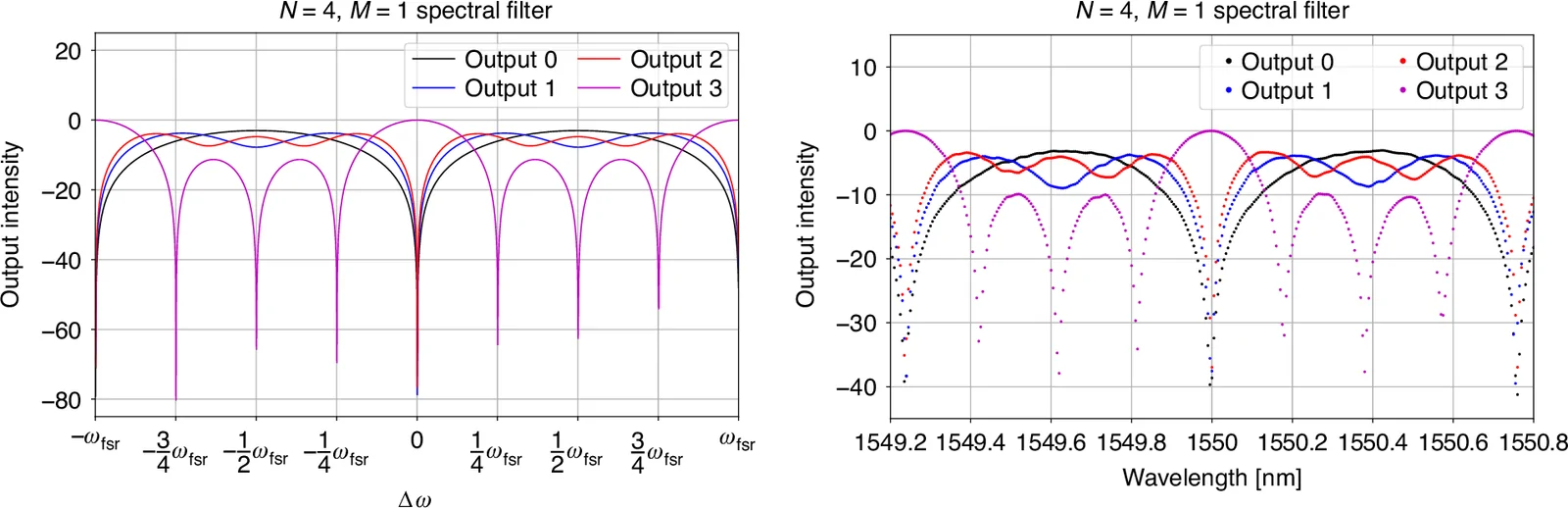
Simulated **(Left)** and measured **(Right)** output spectra of a ${\rm{N}}=4$, ${\rm{M}}=1$ spectral filter which has been programmed via self-configuration. The output spectra demonstrate that light at the central angular frequency ${{\rm{\omega }}}_{0}$ corresponding to a central wavelength ${{\rm\lambda }}_{0}=1550\,\mathrm{nm}$ has been collected to the ${\rm{q}}=3$ output waveguide and has been completely rejected from the remaining outputs. We measure rejection of the central wavelength from the remaining output channels with over 35 dB of contrast and a free spectral range of 0.78 nm

The analytical and experimental output spectra given the previously discussed conditions are plotted in Fig. 3. While the analytical expressions in this work are given in terms of angular frequency, by convention the experimental results are expressed in terms of an equivalent wavelength. The single-layer filter characterized in Fig. 3 has been configured from a larger, multi-layer mesh by setting all subsequent layers to the transparent bar states. For this device, we observe that the transmission to the ${\rm{q}}=3$ output waveguide contains all of the power associated with the pilot source at ${{\rm{\omega }}}_{0}$, corresponding to a ${\lambda }_{0}=1550\,\mathrm{nm}$.

Consequently, none of the power associated with the pilot source is transmitted to the remaining outputs. We measure that light at the center wavelength has been rejected from the remaining output waveguides with contrasts of 40 dB, 38 dB, and 37 dB for ${\rm{q}}=0$, ${\rm{q}}=1$, and ${\rm{q}}=2$ respectively.

An experimental FSR of 95 GHz (0.76 nm) is recorded compared to the designed 100 GHz (0.8 nm). We attribute the 5% error to process variability of the single-mode waveguide cross section, resulting in discrepancies between the modeled and experimental group index. Regardless of such process variations, the center wavelength of the filter has been programmatically optimized to filter light of an arbitrary wavelength, determined only by the choice of pilot source.

An additional dynamic, wavelength-independent phase profile may be introduced using programmable phase shifters to perturb the static phase profile generated by the array of waveguide delay lines. This dynamic phase profile may be generated either using the ${{\rm{\phi }}}_{00}$, ${{\rm{\phi }}}_{01}$, and ${{\rm{\phi }}}_{02}$ phase shifters in the ${\rm{m}}=0$ layer of the photonic mesh or the ${{\rm{\phi }}}_{0}$, ${{\rm{\phi }}}_{1}$, and ${{\rm{\phi }}}_{2}$ phase shifters of the power splitting sub-circuit. Whether this phase profile is introduced prior to or following the array waveguide delay lines is of no consequence. By programming these phase shifters to introduce a linear tilt in addition to the phase shifts determined by self-configuration, the center wavelength of a preconfigured filter function may be shifted. The programmable linear phase profile is defined by the terms ${{\rm{e}}}^{-j2\pi \gamma p}$. By adjusting the value of gamma between 0 and 1, the center wavelength of the preconfigured filter function may be tuned continuously across an FSR.

**Fig. 4:**
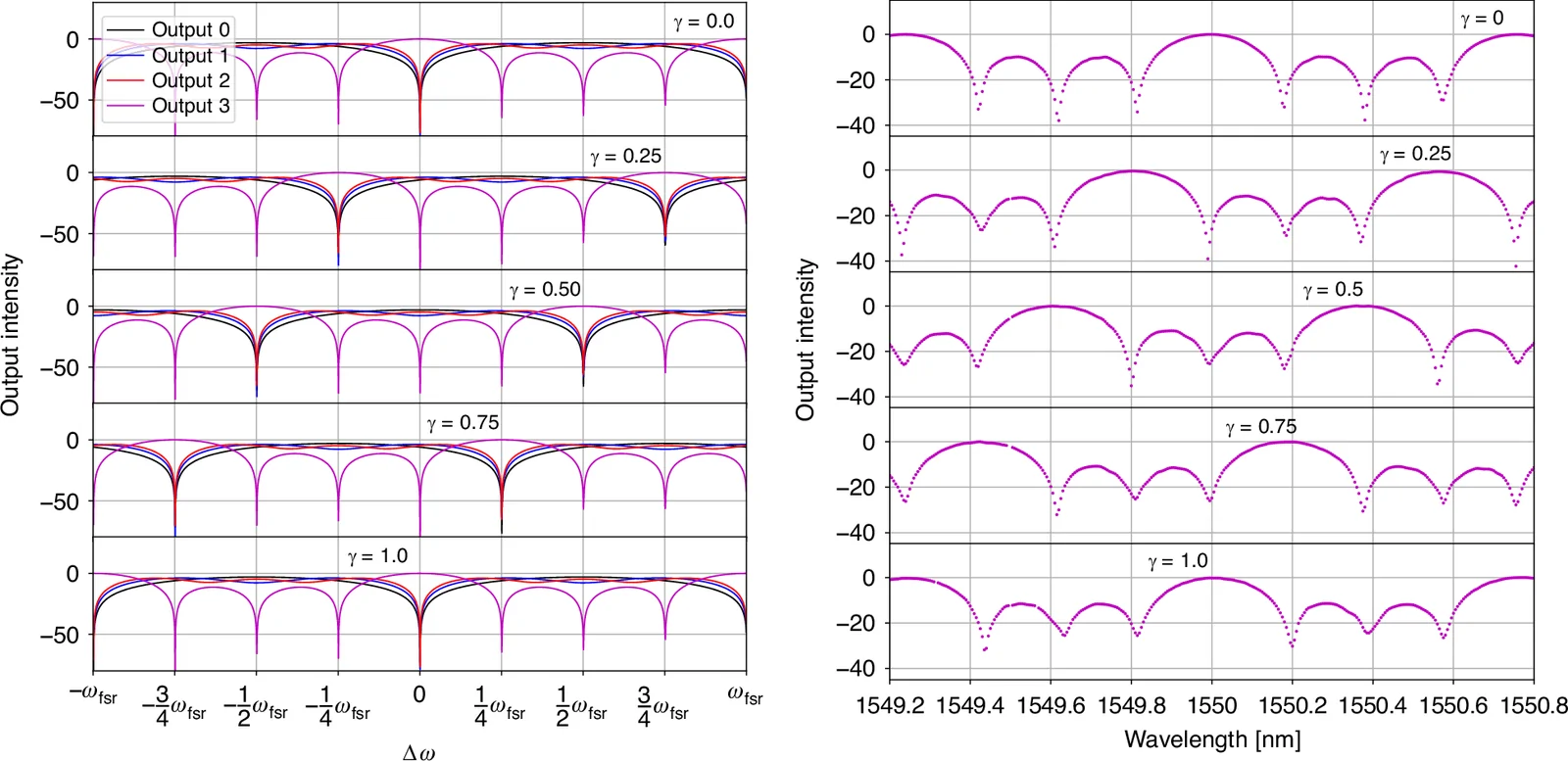
Simulated **(Left)** and measured **(Right)** output spectra of a ${\rm{N}}=4$, ${\rm{M}}=1$ spectral filter while applying an additional phase profile ${{\rm{e}}}^{-j2\pi \gamma p}$ for different values of ${\rm{\gamma }}$. By adjusting the value of ${\rm{\gamma }}$ continuously between 0 and 1, a preconfigured filter function may be tuned continuously across an FSR

This principle is demonstrated for a ${\rm{N}}=4$, M=1 spectral filter for a selection of ${\rm{\gamma }}$ values in Fig. 4. The ability to modulate the center wavelength of a preconfigured filter function is a prerequisite for applications such as correlation spectroscopy used in environmental and biomedical sensing^15^.

It can be seen in Figs. 3 and 4 that the output spectrum of the ${\rm{q}}=3$ waveguide contains ${\rm{N}}-1=3$ nulls separated evenly across the FSR by $\frac{{{\rm{\omega }}}_{{\rm{fsr}}}}{N}=$ 23.75 GHz (0.19 nm). Input light at these given frequencies generate input vectors to the photonic mesh which are all mutually orthogonal to the input vector generated by the initial pilot source with angular frequency ${{\rm{\omega }}}_{0}$. By orthogonality and the fact that any transfer function generated by the photonic mesh is linear and unitary, the filter function generated by the first layer of the mesh will not route any light from wavelengths corresponding to these orthogonal input vectors to the output waveguide ${\rm{q}}=3$. At each of these nulls, light has been rejected from the ${\rm{q}}=3$ output waveguide with a contrast between 28 dB and 38 dB. These nulls determine the optimal wavelength grid, where minimal crosstalk between adjacent channels may be achieved. This kind of frequency response is characteristic of arrayed waveguide grating filters; as such, our circuit can emulate such devices.

It can further be observed from Fig. 3 that outputs ${\rm{q}}=1$, 2, and 3 have multiple transmission peaks with varying relative amplitudes and frequency spacings. The relative amplitude and frequency spacing are functions of the phase shifter settings of the remaining layers of the photonic mesh. These parameters may be adjusted dynamically and arbitrarily, enabling complex functionalities such as intra-FSR pulse shaping. Were this device to act as the wavelength-selective component of an external cavity laser, these multiple transmission peaks may be tuned to enable multimode lasing within a single FSR.

The self-configuration algorithm has effectively generated a spectral filter centered at ${{\rm\lambda }}_{0}=1550\,\mathrm{nm}$ for which all light associated with the pilot source will be routed to the ${\rm{q}}=3$ waveguide and rejected from the remaining outputs. Each additional layer of the mesh may generate an additional independent filter function by applying the same self-configuration algorithm with an independent pilot source. This is such that our ${\rm{N}}=4$ by ${\rm{M}}=3$ spectral filter may reject 3 arbitrary wavelengths from the ${\rm{q}}=0$ output waveguide.

**Fig. 5:**
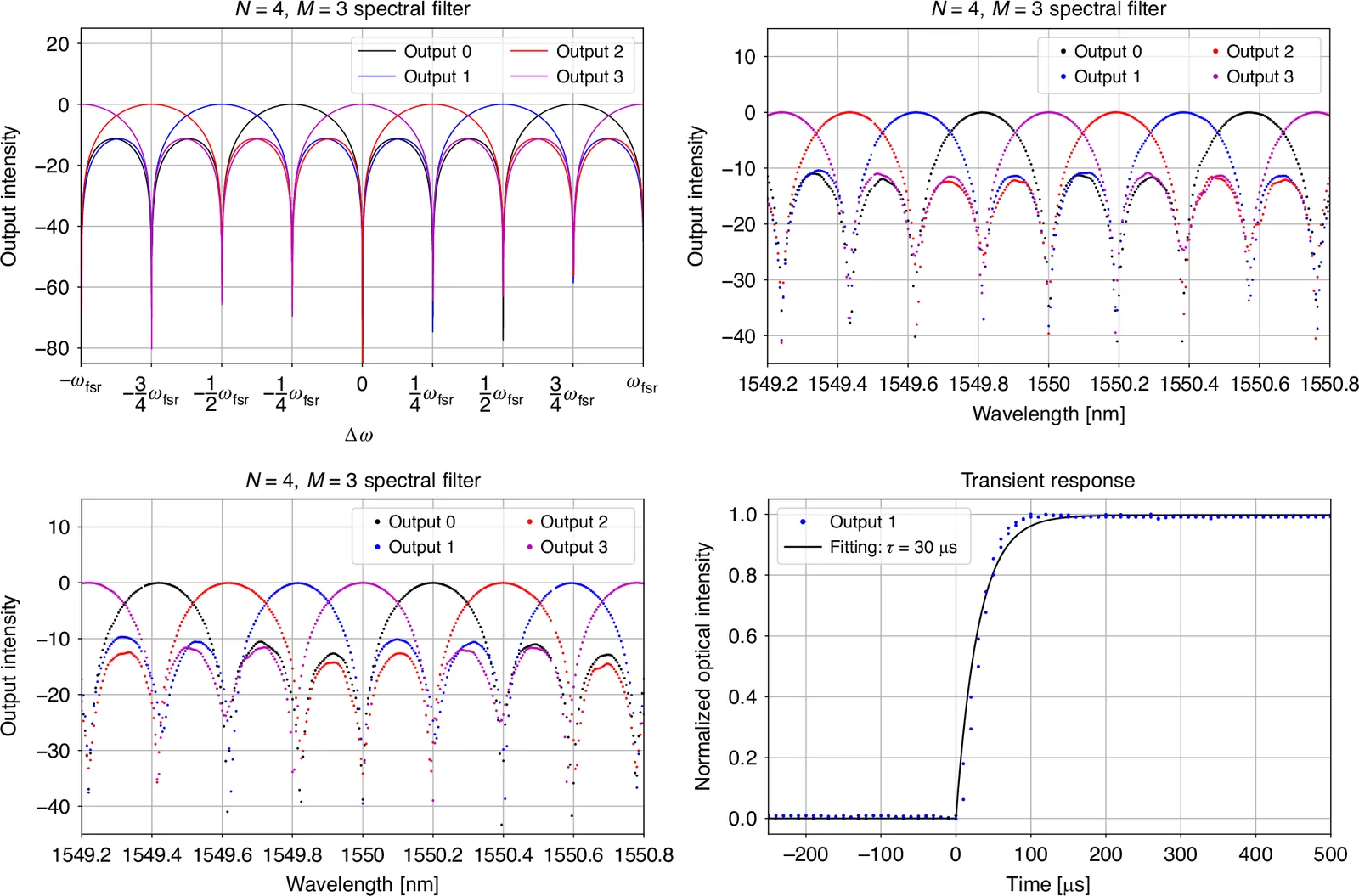
**(Top Left)** Simulated output spectra of a ${\rm{N}}=4$, ${\rm{M}}=3$ spectral filter where each layer of the mesh has been programmed via self configuration to reject a frequency associated with the nulls of each other layer of the mesh. The resulting spectra produce high transmission for a single channel of the generated frequency grid while maintaining high rejection of each other channel. **(Top Right)** The chosen wavelengths of interest are ${{\rm{\lambda }}}_{0}=1550\,{\rm{nm}}$, ${{\rm\lambda }}_{0}=1550\,\mathrm{nm}$, and ${{\rm\lambda }}_{1}=1550.19\,\mathrm{nm}$. **(Bottom Left)** The wavelengths of interest have been reordered here as ${{\rm\lambda }}_{2}=1550.38\,\mathrm{nm}$, ${{\rm\lambda }}_{0}=1550\,\mathrm{nm}$, and ${{\rm\lambda }}_{2}=1550.57\,\mathrm{nm}$ to demonstrate that the devices capacity to route any wavelength channel to any output. **(Bottom Right)** Measured transient response of output waveguide 1 when switching between two preconfigured filter functions

By choosing the frequency of subsequent pilot sources to correspond with the nulls of the first layer of the mesh, self-configuration will result in shifted copies of the same transfer function. We demonstrate this principle for a programmable filter with ${\rm{N}}=4$ inputs and a complete triangular mesh${\rm{M}}={\rm{N}}-1$. Figure 5 displays the resulting transmission functions for a filter configured to this grid.

Here the pilot sources have been chosen to have wavelengths corresponding to the nulls at ${{\rm{\lambda }}}_{0}=1550{\rm{nm}}$, ${{\rm\lambda }}_{0}=1550\,\mathrm{nm}$, and ${{\rm\lambda }}_{2}=1550.38\,\mathrm{nm}$. Though only three pilot sources have been used to configure this filter, we observe that the remaining output channel exhibits an identical shifted transmission function centered on the remaining null with a corresponding central wavelength of 1550.57 nm. Here, each output waveguide achieves high transmission for a single wavelength of interest while maintaining high rejection for the central wavelengths of each other output waveguide. This performs the function of a deep wavelength division demultiplexer, similar to an arrayed waveguide grating filter, and exhibits crosstalk levels between −25 dB and −40 dB. The frequency grid of this device may be arbitrarily shifted across and FSR as given by ${{\rm{\omega }}}_{0}+{{\rm{\omega }}}_{{\rm{fsr}}}[{\rm{\gamma }}+\frac{{\rm{p}}}{N}]$. Each output of the filter can be configured to have unity transmission for a single channel on this frequency grid while maintaining high rejection of each other channel.

In typical DWDM systems, a particular wavelength channel is fixed to a given output waveguide of the device, creating a single wavelength to single output mapping^1^. That is not the case for the architecture discussed here. A particular wavelength channel will be coupled to a given output waveguide based solely on the order in which pilot sources are used during the self-configuration process. Such a device is capable of generating an any wavelength to any output mapping on its respective wavelength grid. We demonstrate this principle by repeating the self-configuration procedure to generate a 0.19 nm wavelength grid with wavelengths of interest: ${{\rm{\lambda }}}_{0}=1550\,{\rm{nm}}$, ${{\rm\lambda }}_{0}=1550\,\mathrm{nm}$, and ${{\rm\lambda }}_{2}=1550.57\,\mathrm{nm}$. The resulting filter functions are displayed in Fig. 5. We observe that similar filter functions have been generated with similar inter-channel crosstalk levels; however, the wavelength bands of interest have been reassigned to new output waveguides.

The phase shifter values determined during self-configuration of any given filter function may be stored digitally and reapplied during future operation of the device. Further, the device can be operated by switching between phase shifter settings for preconfigured filter functions to introduce transient intensity responses at each of the output waveguides. We demonstrate this by switching between the two preconfigured filter functions, which each generate a 0.19 nm wavelength grid. Figure 5 displays the normalized optical intensity measured at output waveguide 1, when a narrow linewidth source with a center wavelength of ${{\rm{\lambda }}}_{1}=1550.57{\rm{nm}}$ has been input to the PIC. Prior to time t = 0 the filter function rejects ${{\rm{\lambda }}}_{1}$ strongly at output waveguide 1. At ${\rm{t}}=0$ the phase shifter settings are updated to the wavelength grid, which channels ${{\rm{\lambda }}}_{1}$ to output waveguide 1. The observed transient response takes the shape of a decaying exponential function${\rm{y}}$ $=1\,-{{\rm{e}}}^{-t/\tau }$, with a time constant ${\rm{\tau }}=30{\rm{\mu }}{\rm{s}}$ which has been determined by a least squared error fitting method. This time constant corresponds to the thermal time constant of the phase shifters used in this work^28^, indicating that the switching speed of such a device is limited by the speed of the underlying phase shifting mechanism.

The inter-channel crosstalk of the DWDM demultiplexer implemented here is consistent with commercial DWDM filter isolation requirements of 25–35 dB. The crosstalk floor in this implementation is likely influenced by the spectral purity of the pilot source used during self-configuration. As the null at a given output deepens, the residual power at the target wavelength may become comparable to contributions from the broadband background of the pilot source, at which point further optimization is limited by the source rather than the phase shifter settings. Improved pilot source spectral purity would therefore be expected to enable deeper nulls and lower inter-channel crosstalk.

**Fig. 6:**
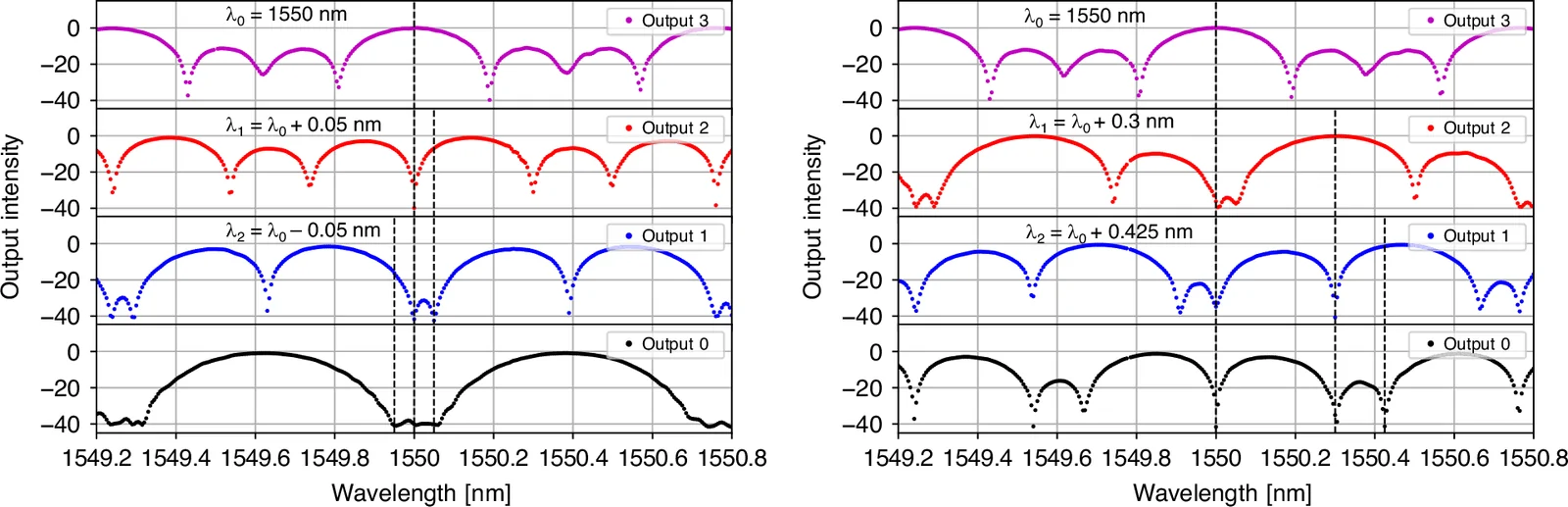
**(Left)** Measured output spectra of a $${\rm{N}}=4$$, M=3 spectral filter where the central wavelength of each filter function has been chosen to be close to each other in the wavelength domain. The corresponding wavelengths of interest here are $\lambda_{0}=1550\;\mathrm{nm}$, $${{\rm\lambda }}_{0}=1550\,\mathrm{nm}$$, and $${{\rm\lambda }}_{1}=1550.05\,\mathrm{nm}$$. **(Right)** Measured output spectra of a $${{\rm\lambda }}_{2}=1549.95\,\mathrm{nm}$$, M=3 spectral filter which has been programmed to apply M filter functions. Here the central wavelength of each filter function has been chosen arbitrarily to be $\lambda_{0}=1550\;\mathrm{nm}$, $${{\rm\lambda }}_{0}=1550\,\mathrm{nm}$$, and $${{\rm{\lambda }}}_{2}=1550.425{\rm{nm}}$$

We further investigate two sub-cases of the programmable filter wherein we have adjusted the center wavelengths of each pilot source. The first sub-case investigates the spectral response of the filter where each of the pilot sources is chosen to be close together in wavelength such that $|{{\rm{\lambda }}}_{{\rm{m}}}-{{\rm{\lambda }}}_{0}| < < {{\rm{\lambda }}}_{{\rm{fsr}}}$. Figure 6 displays the results of a filter configured using pilot sources set to 1549.95 nm, 1550.00 nm, and 1550.05 nm. We observe that in this case, rather than each pilot source being discretely filtered, a rejection band forms around the wavelength range of interest. The configuration demonstrated in Fig. 6a targets a single objective: implementing a wideband deep-rejection filter on output channel 0, which achieves approximately 40 dB of rejection depth over a 0.1 nm bandwidth, which represents ~13% of the FSR. Due to the unitary nature of the matrix transformation of the photonic mesh, optical power not routed to output 0 is conserved and distributed among the remaining ports by energy conservation. In the most conservative interpretation, the remaining outputs may be regarded as dump ports. The resulting differences

in absolute transmission between output ports are therefore expected by design and do not reflect on the performance of the filter at output 0. However, the phase shifter configuration simultaneously defines independent coherent interference conditions at every output port, each implementing its own filter function: output channel 3, for example, simultaneously implements a bandpass filter as a potentially useful byproduct of the same configuration.

The second sub-case allows the choice of center wavelengths for the pilot sources to be chosen arbitrarily over an FSR of the filter. As an example, Fig. 6 displays the spectra from each output waveguide of the filter when the pilot wavelengths have been chosen with no relation. The center wavelengths of each pilot source are chosen to be ${{\rm{\lambda }}}_{0}=1550{\rm{nm}}$, ${{\rm\lambda }}_{0}=1550\,\mathrm{nm}$, and ${{\rm\lambda }}_{2}=1550.425\,\mathrm{nm}$. Each filter is configured to reject its corresponding pilot source from transmitting to the remaining output waveguides. This is such that ${{\rm{\lambda }}}_{0}$ is rejected from the ${\rm{q}}=0$, ${\rm{q}}=1$, and ${\rm{q}}=2$ output waveguides, ${{\rm{\lambda }}}_{1}$ from the ${\rm{q}}=0$ and ${\rm{q}}=1$ output waveguides, and ${{\rm{\lambda }}}_{2}$ from the ${\rm{q}}=0$ output waveguide. We observe that each wavelength has been rejected from the corresponding channels with a contrast between 35 dB and 40 dB. In both of these sub-cases, all three wavelength bands associated with the pilot sources used in self-configuration have been well rejected from the ${\rm{q}}=0$ output waveguide regardless of the existing frequency grid.

**Fig. 7:**
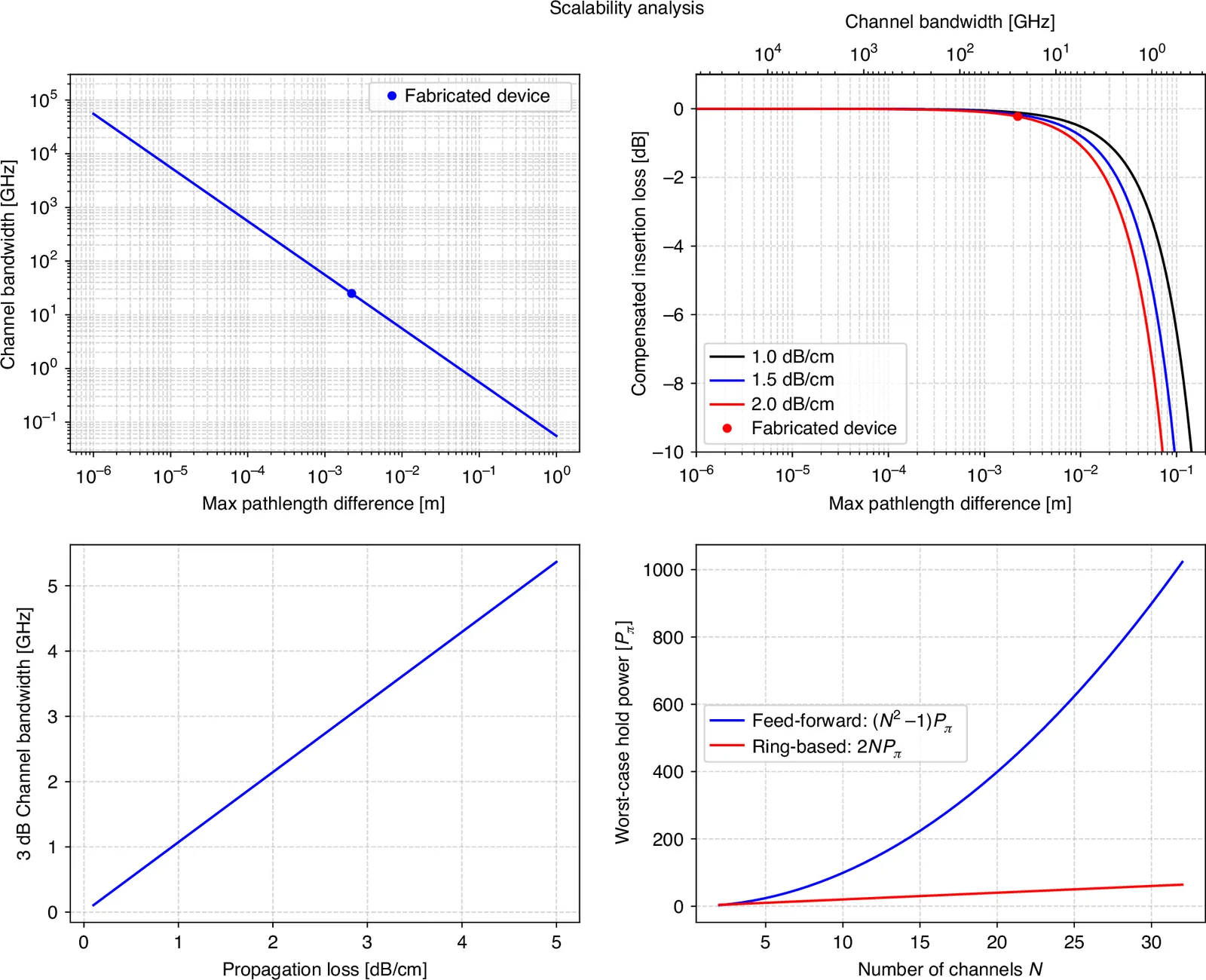
**(Top Left)** Modeled passband channel bandwidth as a function of the maximum pathlength difference in the array of waveguide delay lines. **(Top Right)** The effective transmission when the programmable power splitter redistributes input power to equalize output power across all N channels, as a function of ${l}_{\max }$ for Si waveguide propagation losses of 1.0, 1.5, and 2.0 dB/cm. **(Bottom Left)** The minimum channel bandwidth that maintains less than 3 dB compensated insertion loss as a function of waveguide propagation loss. **(Bottom Right)** Worst case power consumption scaling analysis normalized by units of ${P}_{\pi }$

Lastly, to quantify the scalability of the architecture, we analyze channel bandwidth, insertion loss, and power consumption for increasingly large programmable spectral filters. The fundamental architectural

parameter governing the achievable channel bandwidth is the maximum path length difference in the array of waveguide delay lines ${l}_{\max }=\left(N-1\right)\Delta l$. In the case of the wavelength division demultiplexer, we can define each channel’s bandwidth approximately as ${BW}=\frac{{FSR}}{N}=\frac{c\left(N-1\right)}{{n}_{g}{l}_{\max }N}$ which scales inversely proportionally to the longest path length difference. Critically, the bandwidth of the architecture exhibits a weak dependence on the number of delay line channels for small *N*, which quickly becomes asymptotic as *N* increases. The top left panel of Fig. 7 shows channel bandwidth as a function of ${l}_{\max }$ on a log–log scale. For the fabricated device in this work *l*_max_ = 2.22 mm, with a corresponding designed bandwidth of 25.0 GHz, consistent with the measured value of 23.75 GHz. The 5% difference reflects group-index variation across fabricated waveguides.

As the maximum pathlength difference ${l}_{\max }$ scales, the differential propagation loss in each delay line grows linearly ${T}_{p}={10}^{-\frac{\alpha p\Delta l}{10}}$, where ${T}_{p}$ is the linear transmission of the ${p}^{{th}}$ delay line and α is the propagation loss of the delay line in dB/cm. Were it not for the programmable power splitter, this would lead to significant deviation from the uniform illumination profile discussed in this work at large path length differences. However, the programmable nature of the architecture enables pre-compensation of these losses via an apodised illumination profile given by 1/${T}_{p}$. The effective transmission for the array of waveguide delay lines is then given by ${T}_{{eff}}=N\mathop{\sum }\nolimits_{p=0}^{N-1}{10}^{-\frac{\alpha p\Delta l}{10}}$. The top right panel of Fig. 7 shows the compensated insertion loss, the effective transmission when the programmable power splitter redistributes input power to equalize output power across all N channels, as a function of ${l}_{\max }$ for Si waveguide propagation losses of 1.0, 1.5, and 2.0 dB/cm.

The 3 dB insertion-loss limit, the bandwidth below which compensation cost exceeds 3 dB, is shown in the bottom left panel of Fig. 7 as a function of propagation loss. For silicon strip waveguides (α ≈ 2.0 dB/cm), this limit corresponds to a channel bandwidth of 2.15 GHz (${l}_{\max }$ = 2.6 cm) and for silicon ridge waveguides (α ≈ 1.0 dB/cm), 1.07 GHz (${l}_{\max }$ = 5.2 cm). By pushing propagation losses lower into the sub dB/cm regime using low-loss platforms such as silicon nitride, sub-GHz bandwidths are achievable with this architecture. These results confirm that, for GHz-range channel bandwidths, the propagation loss penalty remains well below 3 dB across all mainstream silicon photonic platforms; meanwhile, the fabricated device in this work operates far from this limit.

Phase errors in the delay lines similarly scale with ${L}_{\max }$, which is fully determined by the target bandwidth; for a fixed bandwidth, ${l}_{\max }$ is independent of N, asymptotic toward $\frac{c}{{n}_{g}{l}_{\max }}$ for large N, so increasing the channel count does not increase the phase error burden. Any residual phase errors from fabrication disorder are directly corrected by the self-configuration protocol, which iteratively tunes the MZI phase shifters to achieve the target filter response without requiring any prior knowledge of fabrication-induced phase deviations. This principle has been experimentally demonstrated in this work, as we are able to generate filter functions that are in very good agreement with the analytical predictions of the architecture, even in the presence of fabrication errors which have led to the observed 5% error in free spectral range.

The power consumption of the feed-forward programmable spectral filter architecture is determined by the two active subcircuits: the power splitter and the programmable photonic mesh. A worst-case hold power for the architecture can be modeled by presuming that each of the N − 1 power splitter phase shifters and all N(N − 1) mesh phase shifters drive a maximum phase value of π radians. In such a case, the total hold power of the system scales as P_H_ = (N − 1)P_π_ + N(N − 1)P_π_ = (N² − 1)P_π_. In comparison, an N-channel ring-based DWDM filter holding each resonance against a worst-case full-FSR detuning requires hold power scaling as 2NP_π_, which is linear with N. The comparison between the power consumption scaling of these two approaches is given in the bottom right panel of Fig. 7. The ratio of these two approaches scales as approximately N/2, so the power disadvantage of the feed-forward architecture grows with channel count.

Despite this power disadvantage, the feed-forward architecture offers capabilities that are fundamentally inaccessible to ring-based filters regardless of their heater configuration. A ring resonator’s spectral transfer function is fundamentally Lorentzian: heaters can shift the resonance wavelength but cannot alter the functional form of the response. The feed-forward architecture instead synthesizes FIR filter responses through coherent superposition of N delayed taps, providing direct access to the full N-tap DFT coefficient space. This enables qualitatively distinct filter functions including the passband, notch, DWDM, and arbitrary rejection demonstrated in this work. In addition, propagation loss affects the two architectures in qualitatively different ways. In a ring resonator, round-trip loss reduces the finesse and directly degrades the Q factor, broadening and shallowing the Lorentzian in a manner that cannot be heater compensated. In the feed-forward architecture, propagation loss manifests as a differential amplitude imbalance across the delay taps, which the programmable power splitter compensates via apodization of the input field. The filter shape is therefore preserved under increasing loss, at the cost of increased overall insertion loss, a favorable tradeoff for any lossy platform. Furthermore, whereas a multi-channel ring system must maintain independent per-channel resonance alignment against thermal drift, the feed-forward architecture responds to thermal perturbations with a global spectral translation that preserves filter shape and can be compensated with a single parameter. Together, these properties- broad reconfigurability, loss-tolerant filter shape preservation, and environmental robustness- define the operating regime in which this architecture is most advantageous relative to resonator-based approaches.

With regard to the thermal stability of the architecture, thermal crosstalk between phase shifters is a transient effect and does not impact hold-state stability. During self-configuration, tuning one phase shifter perturbs neighboring elements transiently; this is mitigated by iterating the procedure until the thermal steady state converges, which requires only a small number of additional passes in practice. The high-resolution spectral measurements in this manuscript were each recorded over several hours with no observable drift, confirming stable hold states on operationally relevant timescales.

## Discussion

We have introduced, modeled, and experimentally demonstrated a photonic integrated circuit that enables fully programmable and reconfigurable spectral filtering on chip. Using self-configuration algorithms, the device can autonomously synthesize a wide range of spectral transfer functions by optimizing each layer of a photonic mesh, allowing targeted rejection or shaping of spectral features. We further demonstrated that these transfer functions can be dynamically adjusted, stored, and recalled, enabling a single PIC to support a broad range of spectral filtering tasks without hardware modification.

This capability enables several important applications, including tunable pump rejection for Raman spectroscopy, continuously tunable and multimode spectral control in external-cavity lasers, and reprogrammable synthetic spectrum generation for correlation spectroscopy. In particular, the ability to shift the central wavelength of a preconfigured filter function, an essential requirement for correlation spectroscopy, is naturally supported by the proposed architecture. We additionally demonstrated dense wavelength-division multiplexing operation with inter-channel crosstalk between −25 dB and −40 dB. Unlike conventional DWDM systems that rely on a static wavelength grid and fixed wavelength-to-port mappings, this device supports dynamic grid translation and arbitrary wavelength remapping to any output channel. This effectively merges programmable filtering and wavelength-selective switching within a single architecture, while providing sufficient degrees of freedom to compensate for fabrication-induced imperfections.

This current *N* = 4 implementation has been intended as a first if its kind, proof-of-concept demonstration. We expect this system to scale to larger implementations and in fact expect the performance in terms of linewidth, crosstalk, and programmability to improve with scale. While it is true that larger scale and longer delay lines may introduce differential loss and larger potential phase errors, both effects may be compensated by the programmable nature of the architecture. The programmable power splitter is specifically implemented for its ability to generate an apodised illumination profile to compensate for any differential propagation losses between the delay lines. As such, any propagation loss imposed by scaling the system will not be prohibitive to the functionality and instead act as an excess insertion loss of the whole system. Any phase error introduced will lead to a deviation from the designed phase profile at the input of the programmable photonic mesh; however, this is an outcome that is already compensated for by the self-configuration algorithm of the programmable photonic mesh. Each of the ϕ phase shifters in the first diagonal line of the photonic mesh may be optimized to compensate for any aberration of the input phase front.

The anticipated limitations for scaling are the required footprint of the waveguide delay lines and photonic mesh as well as the total propagation loss of the system after compensation for the differential loss as discussed above. The required footprint of the delay lines may be mitigated by employing waveguide spiraling techniques such that the area consumed by each waveguide delay line scales approximately linearly with the length of the delay line. Meanwhile, the depth of an N-channel triangular photonic mesh scales linearly with the number of channels and the size of an individual MZI. The acceptable amount of propagation losses will vary by application; however, this propagation loss does not impact the functionality of the device to first order. These two limitations are practical and can trade off amongst one another by switching to low confinement platforms such as silicon nitride, which provide lower propagation losses at the cost of a larger footprint. Moreover, there is no theoretical upper bound for the number of channels, provided there is an application that deems the footprint and propagation loss acceptable.

Our demonstration serves a dual purpose: it provides a practical proof-of-concept implementation employing thermo-optic phase shifters, while also establishing an architectural demonstration of principles that are applicable to any platform and actuation method. The thermo-optic phase shifters used in this work were chosen for their reliability and availability in commercial multi-project wafer processes; however, they present bottlenecks in terms of power consumption, thermal crosstalk, and actuation speed. Each of these limitations of the system may be improved through the implementation of plasma-dispersion, micro-electromechanical (MEMS), piezo-electric, or electro-optic phase shifters. While each of these actuation methods introduce their own practical trade-offs in terms of insertion loss, complexity, yield, power consumption, switching speed, and length, there is no physical limitation preventing this architecture from using any of them.

In this demonstration, we observe a discrepancy between the measured and modeled free spectral range of the filter, likely caused by variation between the modeled and experimental group index of the waveguides. This discrepancy represents one of the only non-programmable degrees of freedom of the system, as the architecture allows us to adjust for the filter shape and center wavelength within an FSR via thermo-optic phase shifters. However, the wavelength dependence of thermo-optic phase shifts is several orders of magnitude weaker than that of the waveguide delay lines, such that the group index may not be easily varied to adjust the FSR. As a direct result, the optimal channel spacing for wavelength division demultiplexing, which is evenly spaced across the FSR and measured at 23.75 GHz cannot be adjusted to achieve the target value of 25 GHz without iterative tapeout cycles. Nonetheless, the device does have the capability to assign arbitrary wavelength grids aside from the optimal wavelength grid defined above, as is demonstrated in Fig. 6. The trade-off of operating at a non-optimal wavelength spacing will be increased crosstalk between channels and decreased transmission at the center wavelength.

It is also worth noting that while we observed a discrepancy between the designed and measured free spectral range, we have not observed variations between the modeled and measured target filter functions. This is a strong indication that the linearity of the phase slope generated by the array of waveguide delay lines is maintained. This may be attributed to the possibility that local fabrication variations are expected to be small, such that the cross section of each of the waveguide delay lines, averaged over the length of the delay lines, has similar group and effective index values to one another, albeit different from the modeled value.

The work presented here offers a new architecture for programmable spectral filtering based on feed-forward photonic meshes. While previous work using resonator-based filters has achieved sharper spectral selectivity in a significantly more compact footprint through resonant enhancement. This approach instead explores a complementary trade-off, prioritizing robustness and programmability over spectral efficiency. Resonator-based filters rely on high-Q feedback, where small variations in effective refractive index produce large changes in spectral response. While this enables compact implementations with sharp roll-off, it also makes such devices inherently sensitive to fabrication-induced phase errors, propagation loss, and environmental fluctuations. In contrast, the feed-forward architecture provides increased tolerance to phase errors and enables compensation of fabrication-induced variations through programming of the mesh. The robustness to environmental fluctuations in this architecture is multi-faceted. First, the programmable elements are based on balanced Mach–Zehnder interferometers, which are nominally insensitive to uniform global temperature shifts. Second, while the delay-line array does experience temperature-dependent phase shifts, the primary effect is a global translation of the filter response in wavelength, rather than a distortion of the filter shape. As a result, environmental drift manifests as a predictable shift that can be compensated, as demonstrated by the ability to shift preconfigured filter functions.

Additionally, in resonator-based filters, achievable sharpness is strongly influenced by round-trip loss, placing stringent requirements on propagation loss and fabrication quality. In the presented architecture, the filter bandwidth scales as ${c}_{0}/({n}_{g}N\Delta L)$, i.e., inversely with the maximum delay. While differential loss between delay paths could in principle degrade performance, the programmable power-splitting network enables apodization of the input field, allowing pre-compensation of loss imbalances and reducing sensitivity to propagation loss in determining the filter response.

Meanwhile, in its current implementation, the feedforward-based architecture requires a significantly larger footprint and power consumption than resonance-based filters. We have performed a worst case power consumption scaling analysis demonstrating that this architecture’s power consumption scales with ${N}^{2}$. In comparison, power consumption for a ring resonator-based approach scales linearly with the number of filter channels. From a system perspective, while resonator-based filters provide high spectral efficiency, their reliance on precise resonance alignment can be a limitation in scenarios involving wavelength drift, thermal variation, or dynamic reconfiguration. The feed-forward approach instead offers a more robust and reconfigurable filtering solution, where spectral responses can be programmably adjusted without dependence on high-Q resonance conditions. We therefore view these approaches as complementary: resonator-based filters are well-suited for compact, fixed-function filtering, whereas the presented feed-forward architecture is advantageous in applications requiring stability, adaptability, and precise control over arbitrary spectral responses.

More broadly, this class of programmable spectral PIC constitutes a spectral analog of reconfigurable photonic meshes that have been extensively studied for spatial mode control. By extending the mesh paradigm into the spectral domain, this work establishes a flexible and scalable approach to on-chip spectral signal processing, with implications for spectroscopy, laser systems, optical communications, and adaptive photonic instrumentation. The architecture and methods presented here provide a foundation for future fully software-defined photonic systems in which spectral functionality is determined by programming rather than fabrication.

## Materials and Methods

Based on the analytical modeling discussed in the previous section, we have designed a programmable spectral filter with a ${\rm{N}}=4$ by ${\rm{M}}=3$ triangular mesh, which has been fabricated by the commercial foundry Advanced Micro Foundry (AMF) Singapore. We have designed this programmable filter for a Silicon-On-Insulator platform with a 220 nm thick silicon device layer, 3 ${\rm{\mu }}{\rm{m}}$ thick buried silicon dioxide layer, and a silicon dioxide top cladding layer.

The single-mode waveguide used throughout the architecture has a cross-section that is 220 nm tall by 500 nm wide. The fundamental quasi-TE mode has an effective and group index of ${{\rm{n}}}_{{\rm{r}}}=2.459$ and ${{\rm{n}}}_{{\rm{g}}}=4.056$, respectively, at a central wavelength of 1550 nm. These values have been simulated using the open-source Eigenmode and FDTD solver EMopt. These waveguides have an approximate propagation loss of 2 dB/cm as provided by the foundry. The coupling of light, both on and off chip, has been managed using surface grating couplers designed to convert between these single-mode silicon waveguides and cleaved single-mode fibers (SMF28). The input and output grating couplers have been characterized to emit at approximately ${12}^{\circ }$ from vertical with approximately −6 dB of loss per coupler. Each of the MZI within the architecture employs large radius of curvature (35 ${\rm{\mu }}{\rm{m}})$ bends with negligible radiative losses to adiabatically transition between beam splitters. Using three-dimensional FDTD simulations, we predict an excess component loss per MZI to be on the order of 0.05 dB independent of propagation losses; however, in practice, this is minimal compared to the expected propagation losses. From these values, we estimate the system losses to be dominated by the input and output coupling losses (12 dB), followed by propagation losses (1.85 dB), and excess component losses (0.35 dB).

The thermo-optic phase shifters used within each MZI are resistive heaters, patterned in a titanium nitride (TiN) layer several microns directly above the waveguiding layer. Each heater is 2 ${\rm{\mu }}{\rm{m}}$ wide by 120 ${\rm{\mu }}{\rm{m}}$ long and has a nominal resistance of 780 Ω. Thermal isolation trenches have been etched through the device layer, buried oxide, and into the silicon substrate on either side of each heater. These trench layers provide a level of mitigation toward thermal crosstalk. Each heating element is independently controlled via a National Instruments digital-to-analog converter (DAC) with 128 analog output channels.

We use an HP 81680 A tunable laser as an input source to define the pilot frequencies throughout this work. The output of the laser is fiber-coupled to a Thorlabs FPC562 manual polarization controller to manage the input polarization state. The output fiber of the polarization controller has been cleaved to ensure good mode matching with surface grating couplers. Both input and output fibers are precisely aligned to their respective grating couplers with a Thorlabs Nanomax 600 series 6-axis stage.

An overhead observation microscope has been assembled to aid coarse alignment between the input/output fibers and their respective grating couplers. A Xenics Bobcat 640 In-GaAs camera is placed in the detector plane of the microscope, enabling in-situ observation of the incident and output fields. This same microscope assembly enables detection of power at the monitor taps used for calibration of the power splitting subcircuit. Fine alignment between input and output fibers is optimized based on throughput power detected at an HP 81531 A photodetector. This same detector is used to measure the output spectra of the programmable filter.

An interface PCB has been designed to route electrical driving signals from the NI DAC to the PIC. A third-party vendor, Silitronics, has been used for the wire-bonding between the PCB bond pads and the PIC bond pads. During the wire-bonding process, the PIC is permanently fixed to the PCB with a thermally conductive adhesive. For thermal management during the operation of the spectrometer, the assembly is secured to an aluminum mount for heat sinking. Additionally, a pocket has been milled into the aluminum mount for a thermo-electric cooling (TEC) unit to be inserted directly beneath the PIC

## Data Availability

Data underlying the results presented in this paper are available from the corresponding authors upon request.
